# Transforming Object Design and Creation: Biomaterials and Contemporary Manufacturing Leading the Way

**DOI:** 10.3390/biomimetics9010048

**Published:** 2024-01-12

**Authors:** Antreas Kantaros, Theodore Ganetsos, Florian Ion Tiberiu Petrescu

**Affiliations:** 1 Department of Industrial Design and Production Engineering, University of West Attica, 12244 Athens, Greece; 2 “Theory of Mechanisms and Robots” Department, Faculty of Industrial Engineering and Robotics, National University of Science and Technology Polytechnic Bucharest, 060042 Bucharest, Romania

**Keywords:** biomaterials, biopolymers, additive manufacturing, 3D design, biomimetic materials, smart materials, self-transforming objects

## Abstract

In the field of three-dimensional object design and fabrication, this paper explores the transformative potential at the intersection of biomaterials, biopolymers, and additive manufacturing. Drawing inspiration from the intricate designs found in the natural world, this study contributes to the evolving landscape of manufacturing and design paradigms. Biomimicry, rooted in emulating nature’s sophisticated solutions, serves as the foundational framework for developing materials endowed with remarkable characteristics, including adaptability, responsiveness, and self-transformation. These advanced engineered biomimetic materials, featuring attributes such as shape memory and self-healing properties, undergo rigorous synthesis and characterization procedures, with the overarching goal of seamless integration into the field of additive manufacturing. The resulting synergy between advanced manufacturing techniques and nature-inspired materials promises to revolutionize the production of objects capable of dynamic responses to environmental stimuli. Extending beyond the confines of laboratory experimentation, these self-transforming objects hold significant potential across diverse industries, showcasing innovative applications with profound implications for object design and fabrication. Through the reduction of waste generation, minimization of energy consumption, and the reduction of environmental footprint, the integration of biomaterials, biopolymers, and additive manufacturing signifies a pivotal step towards fostering ecologically conscious design and manufacturing practices. Within this context, inanimate three-dimensional objects will possess the ability to transcend their static nature and emerge as dynamic entities capable of evolution, self-repair, and adaptive responses in harmony with their surroundings. The confluence of biomimicry and additive manufacturing techniques establishes a seminal precedent for a profound reconfiguration of contemporary approaches to design, manufacturing, and ecological stewardship, thereby decisively shaping a more resilient and innovative global milieu.

## 1. Introduction

The technology of 3D (three-dimensional) printing has revolutionized manufacturing and design, enabling the creation of complex and customized objects with ease. Building upon this transformative technology, the emergence of 4D (four-dimensional) printing has taken additive manufacturing to the next level [1]. Unlike traditional static 3D printing, 4D printing introduces the dimension of time, allowing objects to transform their shape, properties, or functionality over time in response to external stimuli. This groundbreaking concept has opened up new frontiers in engineering, materials science, and robotics, offering unprecedented opportunities for innovation and application [2]. 

At its core, 4D printing encompasses the integration of smart materials that can undergo controlled and programmed shape changes or property alterations when triggered by specific stimuli. These stimuli can range from temperature variations, humidity, light, or even mechanical forces. The materials used in 4D printing possess the remarkable ability to respond to these external cues, initiating a transformation process that leads to dynamic and adaptive behavior [3]. Temporal integration in 4D printing introduces the dimension of time, enabling dynamic transformations and shape changes in printed objects over time in response to various stimuli like temperature, light, or moisture. This evolution significantly expands production possibilities compared to static 4D printing. Benefits of temporal integration in 4D printing include increased complexity and functionality, adaptive and responsive properties, and enhanced customization as well as functional evolution [3]. Static 4D printing involves objects that have predefined, fixed transformations or shape changes. Temporal integration surpasses this by introducing a time-based element, allowing for continuous or triggered changes. While static 4D printing is remarkable, temporal integration elevates its capabilities by offering more dynamic and adaptive structures [3]. 

The fundamental principle behind 4D printing lies in the precise design and fabrication of smart materials with the desired shape-changing properties. Biomimetic smart materials utilized in 4D printing showcase dynamic adaptability, responding to diverse external cues in a way akin to natural responsiveness. These materials react to stimuli like temperature shifts, humidity fluctuations, pH changes, light exposure, or specific chemicals [4]. Triggered by these factors, they undergo alterations, resulting in shape modifications, expansions, contractions, or property changes. By emulating biological responsiveness to environmental cues, these biomimetic materials enable adaptable and dynamic behaviors in 4D printing, fostering applications across fields such as medicine, robotics, and architecture. 

Smart material selection stands as a critical determinant for achieving desired shape-changing characteristics in 4D printing. The choice of smart materials significantly influences the object’s responsiveness to stimuli and its ability to undergo precise transformations over time. Factors such as the material’s inherent properties, including shape memory, responsiveness to specific triggers (like temperature, light, or moisture), durability, and compatibility with the printing process, profoundly impact the efficacy of shape changes [4]. Moreover, considerations regarding the intended application and environmental conditions further shape the selection process, emphasizing the pivotal role of smart material choice in determining the success and functionality of 4D-printed objects. 

Notably, the development of shape-shifting materials has revolutionized the landscape of 4D printing. One prominent example is the utilization of smart materials, such as hydrogels and shape memory polymers, which can transform their shapes in response to external stimuli like temperature, light, or moisture [4]. Researchers have successfully demonstrated the creation of intricate structures that can self-assemble or morph into predetermined shapes, showcasing the potential for applications in various fields, including biomedical devices, aerospace components, and flexible electronics. Additionally, the integration of multi-material and multi-scale 3D printing techniques has led to the production of complex, functional, and customizable products, from light-weight, high-performance automotive parts to intricate, patient-specific medical implants, underscoring the versatility and potential of additive manufacturing in modern technology [5]. 

These materials can be classified into two main categories: shape memory materials and stimuli-responsive materials [6,7]. Shape memory materials have the capacity to “remember” a specific shape and return to it when activated by an external stimulus. Stimuli-responsive materials, on the other hand, can undergo reversible changes in their properties or shape when exposed to specific triggers [8,9]. To realize the full potential of 4D printing, a multidisciplinary approach is essential. Researchers and engineers draw from fields such as materials science, mechanical engineering, computer science, and design to develop innovative strategies for material selection, design optimization, and fabrication techniques [10,11]. Computer modeling plays a pivotal role in forecasting and optimizing 4D-printed structures by simulating and predicting their dynamic behaviors and shape changes over time [12]. Through advanced computational algorithms, these models can simulate the response of materials to different stimuli, allowing for the prediction of structural transformations. By analyzing various parameters such as material properties, environmental conditions, and design intricacies, computer models can optimize the printing process, predict how structures will morph or adapt, and fine-tune designs to regulate and control specific shape changes. This enables a more precise and efficient creation of 4D-printed objects with tailored and regulated dynamic behaviors [13]. 

Biomimetic smart materials epitomize a scientific endeavor rooted in nature’s teachings. These materials, shaped by a profound understanding and emulation of the intricate architectures, adaptive behaviors, and responsive mechanisms inherent in diverse biological systems, span the vast spectrum from macroscopic entities like plants and animals to the microscopic domains of microbes and cellular structures [14]. This scientific pursuit involves an exhaustive exploration of nature’s evolutionarily refined strategies developed over billions of years. Scientists aim to decipher and replicate these exceptional material properties and functionalities previously beyond the scope of conventional methodologies. By meticulously studying nature’s design principles, biomimetic smart materials pave the way for innovations that not only imitate but also surpass the efficiency and adaptability ingrained in natural systems [15]. 

One of the fundamental objectives driving the development of biomimetic smart materials lies in emulating nature’s remarkable adaptability and responsiveness to environmental stimuli [16]. Across various biological systems, there exists a pervasive capacity to sense and dynamically react to alterations in temperature, humidity, light exposure, pressure, and chemical cues [17]. Integrating these inherent responsive capabilities into synthetic materials represents a scientific pursuit aimed at creating innovative materials capable of real-time adaptation, morphing, or self-healing, mirroring the intricate mechanisms observed in living organisms [18]. This scientific pursuit involves a multidisciplinary approach, encompassing materials science, bioengineering, and nanotechnology, among other fields, to replicate and harness the dynamic responsiveness witnessed in biological systems. Researchers meticulously study and replicate these natural mechanisms, seeking to imbue synthetic materials with similar adaptive properties for applications from biomedical devices to advanced engineering and beyond. 

The potential applications of biomimetic smart materials are vast and diverse. From engineering to medicine, robotics to architecture, these materials hold promise for enhancing the performance, sustainability, and functionality of various systems [19,20,21,22]. They offer new opportunities for designing advanced prosthetics, developing self-repairing infrastructure, creating adaptive textiles, fabricating responsive sensors, and revolutionizing energy harvesting and storage [23,24,25,26,27]. In example, the pioneering work of Voronkina et.al. [28] discusses the intricate structure of the Aphrocallistes beatrix spong while looking into the fundamental principles of bioarchitecture, aligning with the biomimetic framework advocated in the broader discourse. The study’s findings, showcasing the positioning of actin filaments within a biosilica-based honeycomb structure, underscore the potential for biomimetic material design [28]. This empirical evidence not only supports the synthesis of biomimetic models through advanced manufacturing techniques like 3D printing but also hints at specialized genetic mechanisms guiding silicate biosynthesis in unique marine environments. Incorporating these insights into the discourse surrounding biomaterials and contemporary manufacturing enhances the paradigm shift towards ecologically conscious design and production practices, ultimately fostering innovative applications that echo nature’s solutions and drive sustainability in object design and fabrication.

Several biomimetic smart materials have emerged as crucial building blocks in the fields of biomimicry and 4D printing [29]. Shape memory alloys (SMAs) are among the most important materials, known for their ability to “remember” and recover their original shape when subjected to certain stimuli [30,31]. They find applications in various fields, including aerospace, robotics, and medicine [32,33]. Another vital material is shape memory polymers (SMPs), which can undergo significant deformation and recover their original shape when triggered by specific stimuli. SMPs are particularly valuable in biomedical applications, such as tissue engineering and drug delivery systems. Additionally, hydrogels, inspired by the water-absorbing properties of biological tissues, have garnered significant attention [34]. These soft, water-swollen materials exhibit remarkable responsiveness to external stimuli, making them ideal candidates for applications in soft robotics, biomedical devices, and sensors [35]. Moreover, electroactive polymers (EAPs) imitate the electrical signaling properties of biological systems, enabling them to undergo shape changes or actuation when an electric field is applied. EAPs have tremendous potential in fields such as artificial muscles, haptic interfaces, and biomimetic sensors. These biomimetic smart materials stand at the forefront of research and development, driving innovations and shaping the future of adaptive and responsive technologies [36,37,38]. 

Biomimetic smart materials represent a pivotal advancement in materials science, with sustainability at their core [39]. By drawing inspiration from nature’s adaptive and self-regulating mechanisms, these materials offer a promising avenue for sustainable innovation. One of their most compelling sustainability aspects lies in their ability to reduce waste and energy consumption. Through self-transformation and adaptability, these materials can optimize their performance in response to changing conditions, thus minimizing the need for constant replacements or interventions [40]. This inherent durability and efficiency align with the principles of a circular economy, where resources are conserved, and environmental impacts are reduced. Additionally, the development of biomimetic smart materials promotes responsible sourcing and production practices, fostering a more environmentally conscious approach to material design. As such, these materials not only offer exciting possibilities for novel applications but also play a significant role in shaping a more sustainable and eco-friendly future [41]. 

**Figure 1:**
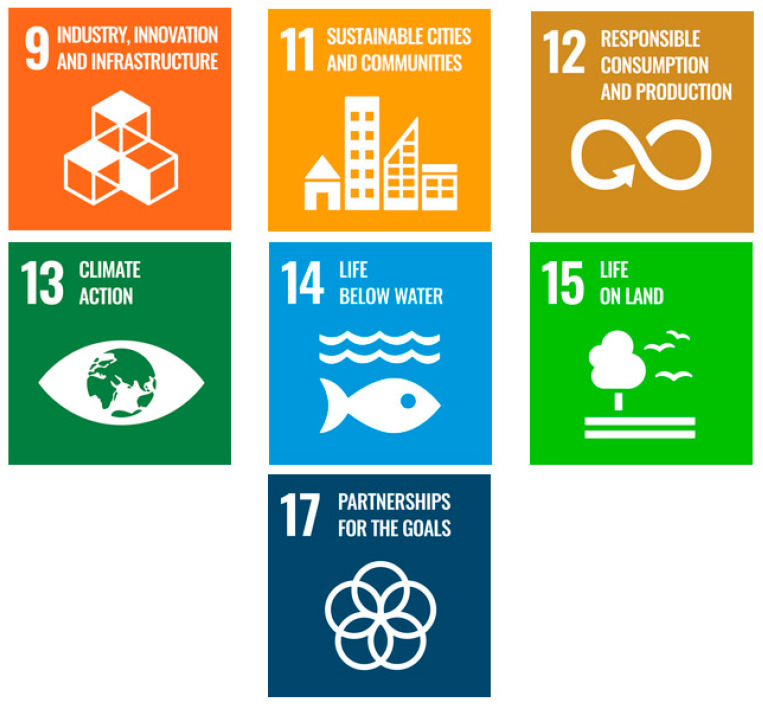
Relevant Sustainable Development Goals (SDGs) proposed by the United Nations [54].

Biomimetic smart materials stand at the forefront of technological advancement, embodying a multifaceted approach that harmonizes seamlessly with several of the key Sustainable Development Goals (SDGs) set forth by the United Nations [42]. By harnessing nature-inspired design principles and cutting-edge technology, these materials play a pivotal role in driving innovation and sustainability across various domains [43,44]. More specifically, as far as “SDG 9—Industry, Innovation, and Infrastructure” is concerned, these materials epitomize innovation, offering groundbreaking solutions that revolutionize industry practices and infrastructure development. Their resource-efficient, self-regulating attributes not only enhance productivity but also contribute significantly to the overarching goal of fostering sustainable industry and infrastructure [45,46]. Also, as far as “SDG 11—Sustainable Cities and Communities” is concerned, in the field of urbanization, biomimetic materials have emerged as transformative agents. They empower the creation of adaptive, resilient urban structures that can thrive amidst evolving environmental challenges. These materials contribute substantially to the sustainable development of cities and the promotion of resilient communities [47,48]. In addition, in compliance with “SDG 12—Responsible Consumption and Production”, biomimetic materials are a characteristic example of responsible consumption and production. By extending the lifespan of products and minimizing waste, they embody sustainable manufacturing and consumption practices. Their innate resource optimization capabilities align closely with SDG 12’s objectives [49]. Regarding “SDG 13—Climate Action”, these materials play a pivotal role in the global endeavor to combat climate change. Through their ability to adapt to changing environmental conditions and reduce energy consumption, they contribute significantly to mitigating climate-related challenges and advancing climate action initiatives [50]. Regarding “SDG 14—Life Below Water” and “SDG 15—Life on Land”, biomimetic materials draw inspiration from the intricacies of ecosystems, thus aligning seamlessly with the preservation and sustainable management of terrestrial and aquatic environments. Their design principles are inherently linked to the goals of conserving biodiversity and maintaining ecological balance [51,52,53]. Lastly, bearing in mind “SDG 17—Partnerships for the Goals”, the development and implementation of biomimetic materials necessitate interdisciplinary collaboration and cooperation. Researchers, industries, governments, and stakeholders unite to pioneer these transformative technologies, thereby nurturing global partnerships and facilitating collective efforts aimed at realizing a sustainable future [54]. In summary, biomimetic smart materials exemplify a holistic and dynamic approach to addressing several pivotal SDGs. Their innovative character, sustainability-driven ethos, and capacity for responsible resource utilization make them instrumental in advancing the global mission for a more sustainable and equitable future, aligning impeccably with the comprehensive development agenda outlined by the United Nations. Figure 1 depicts the aforementioned relevant Sustainable Development Goals (SDGs) proposed by the United Nations.

Challenges associated with biomimetic smart materials for 4D printing arise from the complexity of replicating the intricate functionalities found in living organisms. One major challenge lies in achieving precise control over material properties and responses, ensuring that the desired shape changes or property alterations occur predictably and reliably. Developing materials with the necessary mechanical, thermal, and chemical properties while maintaining responsiveness to stimuli requires a deep understanding of material science and biology. Additionally, scalability and compatibility with existing 4D printing techniques pose challenges, as manufacturing processes need to be optimized for large-scale production without compromising the materials’ functionality. Moreover, long-term durability and stability of biomimetic smart materials remain critical concerns in ensuring their practical viability and longevity in real-world applications [55]. 

However, the challenges of biomimetic smart materials also present significant opportunities for innovation and advancement. The ability to replicate and harness the adaptability and self-regulating mechanisms of living organisms opens up new possi-bilities for engineering design. By leveraging biomimicry principles, researchers can develop materials that exhibit enhanced functionality, responsiveness, and resilience. These materials have the potential to revolutionize fields such as healthcare, robotics, architecture, and more. Biomimetic smart materials enable the creation of self-transforming objects with unprecedented capabilities, from shape-changing structures to programmable soft robots. Furthermore, they offer the prospect of sustainable and eco-friendly solutions by drawing inspiration from nature’s efficient and resource-conserving systems. The advancement of biomimetic intelligent materials within the realm of 4D printing not only affords avenues for scientific and technological progress but also aligns with the overarching objective of fostering enhanced adaptability and intelligence in our environment. 

## 2. Biomimetic Smart Materials

### 2.1. Shape Memory Alloys (SMAs)

Shape memory alloys (SMAs) are a remarkable class of biomimetic smart materials that exhibit the ability to remember and recover their original shape after defor-mation when subjected to specific stimuli. These materials have gained significant attention due to their unique shape memory effect, which is derived from a reversible phase transformation between austenite and martensite crystal structures. This remarkable property allows SMAs to undergo substantial deformations and then revert to their original shape when triggered by changes in temperature, stress, or magnetic fields [56]. 

One of the most notable features of SMAs is their high capacity for recoverable strain, which can exceed several hundred percent. This exceptional property makes them ideal for applications requiring large actuation capabilities, such as in actuators, valves, and micro-robotic systems [57]. SMAs can deliver precise and controlled movements, enabling them to mimic the motion and functionality of biological systems. Their excellent me-chanical properties, including high strength, fatigue resistance, and good damping characteristics, contribute to their suitability for various engineering applications [58]. 

SMAs have wide-ranging applications across numerous fields. In the aerospace industry, SMAs are utilized in adaptive wing structures, morphing airfoils, and de-ployable space structures, where their ability to undergo shape changes in response to environmental conditions provides significant advantages in aerodynamic performance and structural optimization [59,60,61]. In the biomedical field, SMAs are used in orthopedic implants, stents, and dental braces due to their biocompatibility, corrosion resistance, and shape memory behavior, enabling minimally invasive procedures and enhanced patient comfort [62]. 

**Figure 2:**
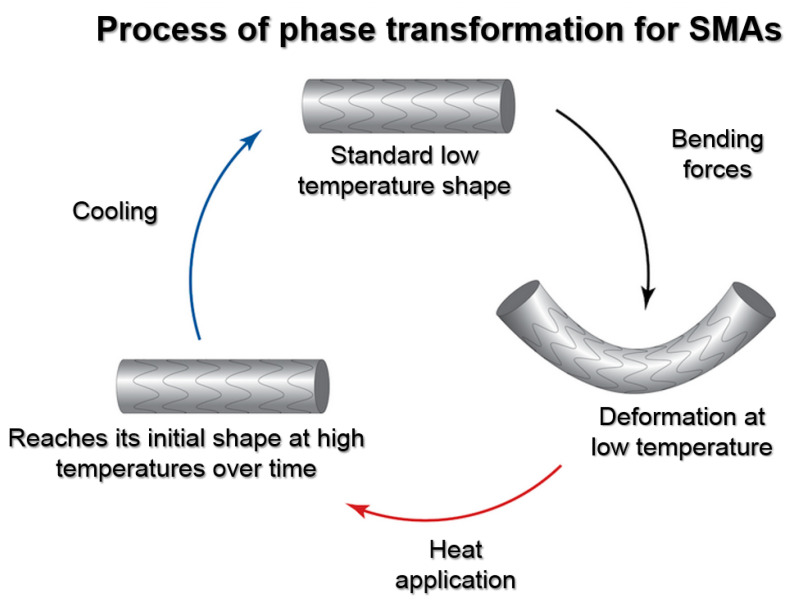
Phase transformation process for SMAs.

Regarding robotics applications, shape memory alloys (SMAs) emulate natural adaptation and mobility through their distinctive properties. With their remarkable shape memory effect and superelasticity, SMAs replicate the adaptive capabilities observed in nature. These alloys have the ability to revert to their original shape after deformation when triggered by specific stimuli like temperature changes, akin to how living organisms adapt to varying conditions [63]. Moreover, their superelastic nature allows SMAs to endure substantial reversible deformations without permanent damage, resembling the elasticity found in biological tissues. This enables robotic components to maintain structural integrity during agile movements, mirroring the resilience and mobility seen in natural organisms. SMAs’ responsiveness to external stimuli, such as temperature or mechanical stress, allows for real-time adjustments in shape and function, contributing to the adaptability and dynamic mobility required in robotics. By leveraging these properties, SMAs serve as efficient actuators, converting thermal or mechanical energy into motion, thereby facilitating the creation of agile and mobile robotic systems that parallel the adaptability and mobility observed in nature [64]. Figure 2 depicts the phase transformation process for SMAs. 

While SMAs offer numerous advantages, challenges exist in their implementation. Designing precise control mechanisms and understanding the intricate thermo-mechanical behavior of these materials requires expertise and thorough characterization. Moreover, issues related to cost, fatigue life, and reliability in long-term applications necessitate ongoing research and development efforts. Nonetheless, with their exceptional shape memory effect and mechanical properties, SMAs continue to captivate researchers and engineers [65]. Their ability to emulate the adaptability and motion found in nature holds immense potential for creating advanced biomimetic systems in fields ranging from robotics and aerospace to medicine and beyond. As research progresses and understanding deepens, the applications of SMAs are expected to expand further, contributing to the development of innovative and intelligent technologies. 

Among the various shape memory alloys (SMAs), nickel–titanium (Ni-Ti) alloys, commonly known as Nitinol, stand out as the most important and widely studied. Ni-tinol exhibits exceptional shape memory properties, high recoverable strain capacity, and excellent biocompatibility, making it a highly versatile and sought-after material [66]. Its unique characteristics, including a wide transformation temperature range, remarkable mechanical properties, and excellent corrosion resistance have led to its extensive use in diverse fields. Nitinol has applications in the aerospace industry for adaptive structures, in the medical field for stents and orthopedic implants, and in robotics for actuators and artificial muscles. The ability of Nitinol to undergo repeated and reversible shape changes with high precision and reliability has positioned it as a key material in the realm of biomimetic smart materials, driving advancements in various technological domains [66]. 

Another significant shape memory alloy (SMA) is copper–aluminum–nickel (Cu-Al-Ni). This ternary alloy exhibits remarkable shape memory behavior and mechanical properties that make it a valuable material for numerous applications. Cu-Al-Ni SMAs possess a two-way shape memory effect, allowing them to recover not only their original shape but also a secondary shape after deformation and subsequent heating or cooling cycles [67]. This property enables complex and multi-step shape transformations, expanding the range of potential applications. Cu-Al-Ni SMAs are particularly suited for precise actuation systems, such as in microelectromechanical systems (MEMs), where their excellent shape memory properties, low hysteresis, and high mechanical stability are advantageous. Additionally, the biocompatibility of Cu-Al-Ni SMAs has led to their utilization in medical devices, including orthodontic wires, endodontic instruments, and vascular stents. The unique combination of shape memory behavior, mechanical properties, and biocompatibility make Cu-Al-Ni SMAs an important material with diverse applications in fields ranging from aerospace and robotics to healthcare and beyond [67]. 

Another shape memory alloy (SMA) worth mentioning is iron-based SMAs, also known as ferrous-based SMAs [68]. Iron-based SMAs offer distinct advantages due to their low cost, abundance, and excellent mechanical properties. These alloys, typically based on iron, can exhibit shape memory and superelasticity, making them suitable for a wide range of applications. Iron-based SMAs have garnered attention in fields such as automotive engineering, where they are used in active suspension systems and crash energy absorbers. Their exceptional damping properties make them valuable for vibration control applications in civil engineering structures and mechanical systems. Furthermore, iron-based SMAs show promise in the biomedical field, with applications in orthodontic wires, surgical instruments, and implantable devices due to their compatibility with the human body. The availability, affordability, and favorable mechanical properties of iron-based SMAs make them an important material for various industrial sectors, contributing to the advancement of shape memory alloy technology. Table 1 lists the most common SMAs, their applications, and their most prominent features [69]. In the following tables (Table 1, Table 2 and Table 3), the specific criteria and metrics used to evaluate attributes such as strength, cost-effectiveness, and biocompatibility vary based on industry standards, research findings, or expert opinions in the fields of materials science or engineering. The assessments are relative rather than absolute, providing a comparative view of these attributes among different materials. 

### 2.2. Shape Memory Polymers (SMPs)

Shape memory polymers (SMPs) are a class of biomimetic smart materials that possess the ability to “remember” their original shape and recover it after being deformed, triggered by specific stimuli such as temperature, light, or pH changes. SMPs are typically composed of a polymer network with the capability to exhibit two distinct states: a temporary shape that can be easily manipulated, and a permanent shape that the material will revert back to when activated. This unique behavior stems from the reversible transitions between the material’s glassy and rubbery states, allowing for significant deformation and shape recovery [70]. One of the remarkable features of SMPs is their tunability, as their mechanical properties and transition temperatures can be tailored by modifying the polymer composition and crosslinking density. This flexibility enables the design and fabrication of SMPs with desired shape memory behaviors suitable for specific applications. SMPs offer advantages such as low weight, low cost, and ease of processing, making them attractive for a number of fields [71]. 

**Figure 3:**
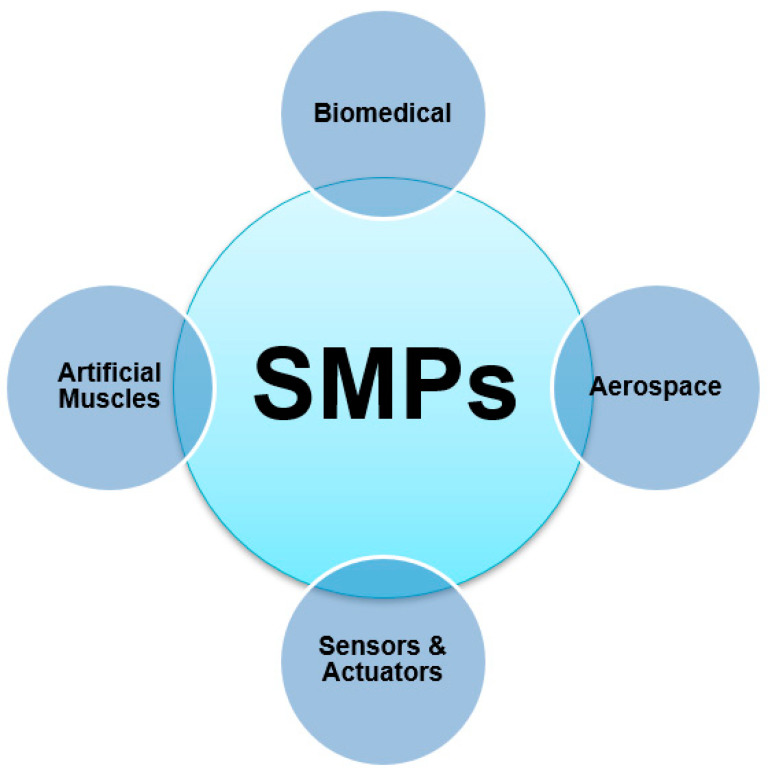
Major application fields for SMPs.

The applications of SMPs span diverse industries. In the biomedical field, SMPs have gained attention for their potential in minimally invasive surgery, tissue engineering scaffolds, drug delivery systems, and shape-adaptive medical devices [72]. SMPs can be designed to respond to body temperature or other physiological cues, allowing for precise, controlled, and localized shape changes in response to the surrounding environment [73]. In the aerospace sector, SMPs find use in morphing structures, deployable systems, and adaptive components, where their shape memory behavior can enable efficient aerodynamic profiles and structural optimization [74,75]. Figure 3 depicts the major fields where SMPs apply.

The practical implementation of shape memory polymers (SMPs) encounters various challenges that ongoing research aims to address. Achieving precise control over shape memory transitions remains a primary hurdle, demanding a comprehensive understanding of how these materials respond to diverse stimuli. Enhancing durability and fatigue resistance stands as another critical area necessitating attention and improvement within SMP research. Researchers are dedicated to unraveling these challenges by delving into advanced methodologies, seeking to grasp the intricate mechanisms governing SMP behavior under different conditions. Moreover, there is a concerted effort towards developing SMPs with multifunctional properties, integrating stimulus-responsive shape memory behavior with capabilities such as self-healing or conductivity [75]. Despite these challenges, the versatile nature of SMPs continues to inspire researchers and engineers. Their unique ability to endure substantial deformation and recover their original shape offers a multitude of possibilities for innovative applications. Ongoing progress in SMP research holds promise for the development of advanced smart materials capable of adapting and responding to changing environments. Researchers are exploring novel material formulations, advanced processing techniques, and predictive modeling approaches to overcome these challenges, aiming to pave the way for next-generation technologies in diverse fields like medicine, aerospace, robotics, and beyond. Through interdisciplinary collaboration and innovative approaches, research endeavors seek to overcome these hurdles and harness the full potential of shape memory polymers in practical applications. 

Among the various shape memory polymers (SMPs), polyurethane-based SMPs hold significant importance and have been extensively studied and utilized in a wide range of applications [76]. Polyurethane SMPs offer excellent shape memory behavior, mechanical properties, and processability, making them highly versatile for engineering applications. Their shape memory effect can be triggered via temperature, allowing for reversible shape changes upon heating or cooling. Polyurethane SMPs find extensive use in fields such as biomedical engineering, where they are employed in shape memory sutures, stents, and tissue engineering scaffolds. Additionally, their biocompatibility, flexibility, and customizable properties make them suitable for drug delivery systems and wearable devices. In industries like aerospace and robotics, polyurethane SMPs are utilized in morphing structures, adaptive components, and soft actuators, using SMPs’ shape-changing capabilities for enhanced performance and functionality. The broad range of applications and the favorable combination of properties make polyurethane-based SMPs the most important and widely utilized among the various types of shape memory polymers [77]. 

Another noteworthy class of shape memory polymers (SMPs) is based on poly-caprolactone (PCL), a biodegradable and biocompatible material [78]. PCL-based SMPs possess shape memory properties that can be triggered by temperature changes. These SMPs exhibit excellent shape recovery and mechanical properties, making them valuable for various applications. In the biomedical field, PCL-based SMPs find use in tissue engineering scaffolds, drug delivery systems, and wound dressings, as they can adapt to the body’s contours and provide controlled release of therapeutics. The biodegradability of PCL allows for the gradual regeneration of tissue over time. Moreover, PCL-based SMPs have also been explored in areas such as soft robotics and textiles, where their shape memory behavior enables the development of adaptable and responsive systems. The versatility and biocompatibility of PCL-based SMPs position them as a significant material for creating smart shape-changing structures and devices with potential applications in diverse fields [79]. By comparing polyurethane-based shape memory polymers (SMPs) and polycaprolactone-based SMPs, distinct features and diverse applications can be found. Polyurethane-based SMPs often exhibit faster shape recovery rates and higher shape fixity compared to polycaprolactone-based ones. They tend to have superior mechanical properties, offering higher strength and toughness, making them suitable for applications requiring robustness, such as in structural components or medical devices. On the other hand, polycaprolactone-based SMPs typically demonstrate greater elongation at their breaking point and are more flexible, enabling them to be utilized in applications demanding higher deformability and shape adaptability, like in soft robotics or biomedical devices where flexibility is paramount [79]. These distinctions in mechanical properties and flexibility allow for tailored applications in various industries, highlighting the versatility of shape memory polymers based on their composition and characteristics. 

Polyethylene-based shape memory polymers (SMPs) represent a notable class of SMPs with distinct advantages and applications [80]. These SMPs are derived from poly-ethylene, a widely used and versatile polymer known for its excellent mechanical properties and chemical resistance. Polyethylene-based SMPs display shape memory be-havior that can be triggered by temperature, enabling reversible shape changes. Their high elasticity and recoverable strain capacity make them suitable for applications requiring large deformations and shape recovery. Polyethylene-based SMPs have found applications in various fields, including automotive engineering, where they are used in automotive components, such as self-repairing bumpers and shape-adaptive panels. Ad-ditionally, in the field of soft robotics, polyethylene-based SMPs are employed in the development of flexible actuators and grippers that can adapt to complex shapes and perform delicate tasks. The robustness, processability, and shape memory properties of polyethylene-based SMPs make them an important material for engineering applications, showcasing their potential to advance technologies in diverse industries. Table 2 depicts the most common SMPs, their applications, and their most prominent features [81].

**Table 2:** Most common SMPs, their applications, and their most prominent features.

SMP Type	Applications	Strength	Cost-Effectiveness	Biocompatibility
Polyurethane-based [76,77]	Biomedical devices, textiles, actuators	Moderate	Moderate	Varies (depends on formulation)
Polyethylene-based [80,81]	Textiles, automotive applications, robotics	Low to moderate	Low to moderate	Generally good
Polyvinyl-based [70,72]	Biomedical devices, textiles	Low to moderate	Moderate	Generally good
Epoxy-based [71]	Aerospace applications, robotics, deployable structures	Moderate to high	Moderate to high	Varies (depends on formulation)
Polycaprolactone [78,79]	Biomedical implants, drug delivery	Low to moderate	Moderate	Generally good
Polyethylene terephthalate (PET) [80,81]	Textiles, packaging, automotive applications	Moderate to high	Moderate to high	Generally good

### 2.3. Electroactive Polymers (EAPs)

Electroactive polymers (EAPs) are a class of materials that possess the unique ability to undergo significant shape changes or actuation in response to electrical stimulation. These polymers imitate the electrical signaling properties of biological systems, enabling them to convert electrical energy into mechanical motion. This remarkable characteristic makes EAPs highly attractive for a wide range of applications, including robotics, sensing, artificial muscles, and biomedical devices [82]. 

**Figure 4:**
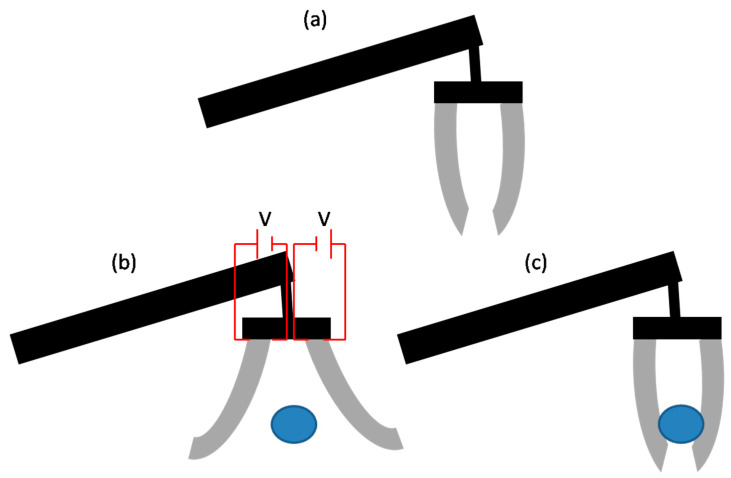
EAP undergoing shape changes in response to electrical stimulation, (**a**) initial position, (**b**) open position, (**c**) closed position.

One of the most well-known types of EAPs is dielectric elastomers (DEs) [83]. DEs consist of a flexible elastomeric material sandwiched between two compliant electrodes. When an electric field is applied, the electrodes attract each other, causing the elastomer to compress or expand. This deformation allows DEs to possess large actuation capabilities and have applications in areas such as soft robotics, where they can replicate natural muscle-like movement and dexterity [83]. Another significant type of EAP is conducting polymer-based EAP, which includes materials like polypyrrole and polyaniline. These polymers exhibit electrical conductivity that can be modulated via an applied electric field [84]. By controlling the doping or de-doping process, the polymer can undergo changes in volume, shape, or mechanical properties. Conducting polymer-based EAPs have been utilized in actuators, sensors, and artificial muscles, offering advantages such as low weight, flexibility, and responsiveness [85]. Figure 4 shows such an EAP undergoing shape changes in response to electrical stimulation.

While EAPs offer great potential, there are challenges that need to be addressed for wider practical implementation. These challenges include improving the materials’ mechanical stability, enhancing the efficiency of energy conversion, and increasing their operational lifespan. Researchers continue to explore new EAP formulations, fabrication techniques, and actuation mechanisms to overcome these obstacles and unlock the full potential of EAPs. 

The field of electroactive polymers holds promise for revolutionizing numerous technological domains, ranging from soft robotics and haptic interfaces to biomedical devices and energy harvesting systems. The ability of EAPs to imitate the electrical behavior of biological systems provides opportunities for the development of advanced, adaptive, and intelligent materials that can respond to electrical stimuli. As research progresses and technologies evolve, electroactive polymers are poised to play a pivotal role in shaping the future of engineering, robotics, and human-machine interfaces. 

Conductive polymer-based EAPs, such as polypyrrole (PPy), represent another significant class of electroactive polymers with intriguing properties [86]. PPy is a conju-gated polymer that exhibits electrical conductivity and can undergo reversible changes in volume and shape in response to electrical stimulation. The doping and de-doping process of PPy, driven by redox reactions, leads to variations in its electrical and me-chanical properties. This makes PPy a promising material for applications such as ac-tuators, sensors, and energy harvesting devices. PPy-based EAPs offer advantages like high responsiveness, mechanical flexibility, and biocompatibility, which make them suitable for emerging fields such as soft robotics, bioelectronics, and biomedical engineering. The tunability of PPy-based EAPs, along with their compatibility with traditional fabrication techniques, holds promise for the development of innovative and efficient electroactive systems that bridge the gap between biological and artificial systems [86]. 

Another noteworthy electroactive polymer (EAP) is the ferroelectric polymer poly(vinylidene fluoride-trifluoroethylene) (PVDF-TrFE) [87]. PVDF-TrFE exhibits piezoelectric properties, meaning it can generate electrical charges when subjected to mechanical stress and, conversely, undergo mechanical deformation when an electric field is applied. This unique property enables PVDF-TrFE to be utilized in various applications, including sensors, actuators, energy harvesting devices, and biomedical applications. PVDF-TrFE-based EAPs offer advantages such as high mechanical flexibility, good chemical stability, and biocompatibility, making them suitable for wearable electronics, human–machine interfaces, and implantable devices. The ability of PVDF-TrFE to convert mechanical energy into electrical energy and vice versa holds tremendous potential for advancing technologies in areas ranging from smart sensing systems to self-powered devices. Table 3 depicts the most common EAPs, their applications, and their most prominent features [87].

**Table 3:** Most common EAPs, their applications, and their most prominent features.

EAP Type	Applications	Strength	Cost-Effectiveness	Biocompatibility
Polypyrrole [86]	Artificial muscles, sensors, actuators	Low to moderate	Moderate	Limited
Polyaniline [84]	Sensors, actuators, electronic textiles	Low to moderate	Moderate	Limited
Ionic Polymer-Metal Composite (IPMC) [82]	Soft robotics, sensors	Low	Moderate	Limited
Dielectric Elastomer [83]	Soft robotics, haptic feedback, medical devices	Low to moderate	Moderate	Limited
Conductive Elastomer [85]	Tactile sensors, wearable electronics	Low to moderate	Moderate	Limited
Ferroelectric Polymers [87]	Energy harvesting, sensors	Low to moderate	Moderate	Limited

## 3. Applications of Biomimetic Smart Materials

### 3.1. Healthcare Field and Regenerative Medicine

As mentioned before, biomimetic smart materials find a wide range of application when used as raw materials in 4D printing processes. The applications of biomimetic smart materials in 4D printing span diverse industries, including healthcare, robotics, architecture, and aerospace. 

In the field of healthcare, biomimetic smart materials are revolutionizing tissue en-gineering and regenerative medicine [88]. By mimicking the extracellular matrix and cellular mechanisms found in natural tissues, 4D-printed scaffolds made from biomimetic smart materials can dynamically adapt to the surrounding environment and guide tissue regeneration. These materials can respond to physiological cues, such as pH or temperature changes, and deliver therapeutic agents with precise control. The ability to create biomimetic structures that mimic the complexity of natural tissues opens up new possibilities for organ transplantation, wound healing, and personalized medicine [88]. 

One specific application lies in the development of 4D-printed scaffolds for tissue engineering [89]. Biomimetic smart materials can replicate the dynamic behavior and mechanical properties of natural tissues, allowing for the creation of scaffolds that closely mimic the extracellular matrix. These scaffolds can respond to physiological cues, such as changes in pH or temperature, to guide and promote cell growth, differentiation, and tissue regeneration. By incorporating shape memory effects or stimuli-responsive behaviors into the scaffolds, researchers can create structures that dynamically adapt to the evolving needs of the tissue, facilitating the healing process [90]. 

**Figure 5:**
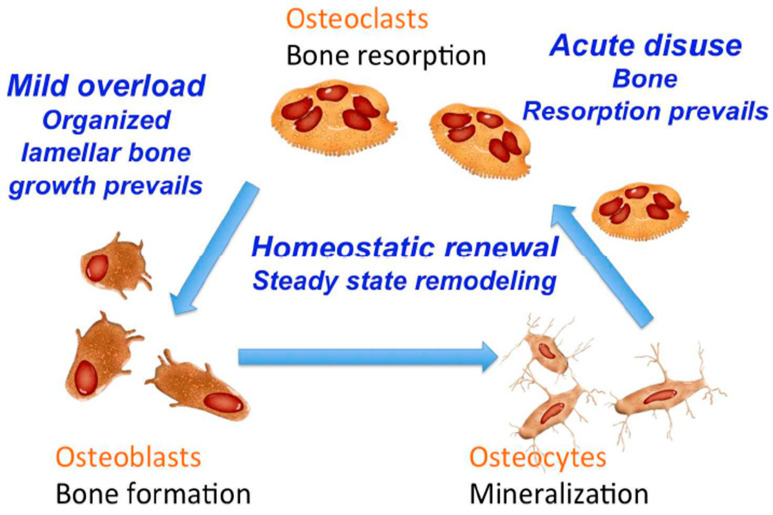
Bone homeostasis mechanism involving osteoblast, osteocyte and osteoclast cells triggered by mechanical stimuli.

In this context, Apicella et. al. described a novel class of biomaterials composed of a hydrophilic polymer matrix infused with nanodiamonds, showcasing distinctive mechanical and biological properties that hold great promise for advanced biomedical applications [91]. This innovative hybrid material [92], created with precise percentages of detonating nanodiamonds and hydrophilic poly (hydroxy-ethyl-methacrylate), represents a groundbreaking approach in scaffold design. It not only addresses the mechanical shortcomings typically associated with hydrogels but also introduces a biomimetic, osteoconductive, and osteoinductive scaffold. The enhanced mechanical strength of these nanocomposites surpasses that of conventional hydrogels, making them suitable for use as osteoinductive coatings on metal trabecular scaffolds. By proposing the application of micro-trabecular metal structures coated with these active biomechanical ceramo-polymeric scaffolds, the paper contributes to the advancement of scaffold fabrication techniques, aiming to replicate the macro- and micro-distribution of bone stresses and deformations in a manner that holds great potential for tissue regeneration and bone growth. Figure 5 depicts the aforementioned bone homeostasis mechanism involving osteoblast, osteocyte and osteoclast cells triggered by mechanical stimuli.

In another instance, Aversa et. al. highlighted the importance of maintaining healthy bones for overall human health and vitality, especially their role in cellular growth and repair [93]. The research focused on developing and testing hybrid biomimetic materials that serve as mechanically stimulating “scaffolds” for promoting early regeneration during implanted bone healing phases. Key contributions included introducing a biomimetic nanostructured osteoconductive material and employing a multidisciplinary approach, combining in vivo, in vitro, and computer-aided simulations. These innovations have enhanced our understanding of bone growth and mechanical loading. In vivo tests in rabbits have demonstrated the effectiveness of mechanical stimulation in promoting mesenchymal tissue development. This research advanced bone regeneration techniques and had significant implications for improving bone-related medical procedures.

**Figure 6:**
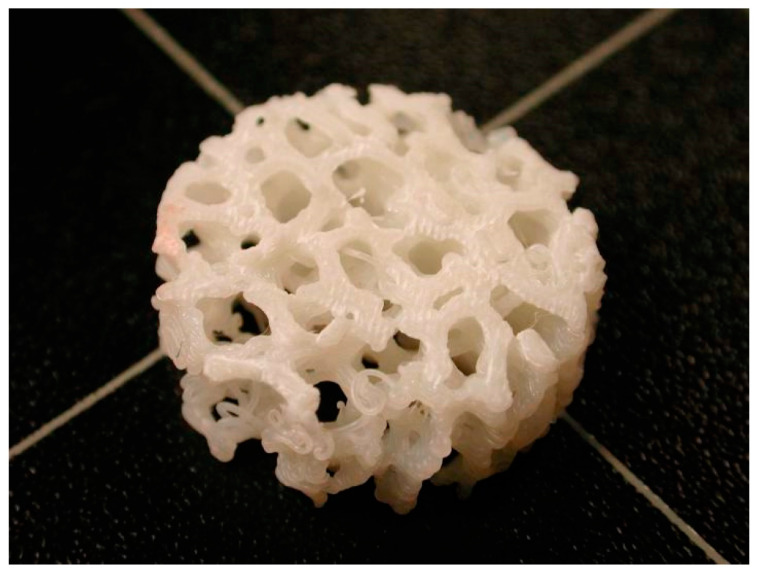
Scaffold structure, designed with biomimicry criteria.

In addition, Aljohani and Desai [94] addressed a critical need in regenerative medicine—custom-engineered scaffolds that closely mimic native tissue physiology. They recognized a key challenge in current manufacturing methods, which is controlled porosity throughout scaffold structures. This research explored the fabrication of tissue engineering scaffolds using 3D printing technology, specifically employing the fused deposition modeling method. Various scaffold designs with different unit-cell configurations and in-fill densities were created by utilizing biomimetic materials. This research underscored the potential for tailoring scaffold structures to balance cellular functionality and load-bearing requirements for specific medical applications by manipulating unit-cell types, infill densities, and pattern orientations. Overall, this study layed a foundational understanding of additive manufacturing technologies for biomedical implants by exploring various process parameters and material properties, contributing significantly to the advancement of tissue engineering and regenerative medicine. Figure 6 depicts such a scaffold structure whose design was created with biomimicry criteria.

Biomimetic smart materials in 4D printing also enable the precise delivery of thera-peutic agents through stimuli-responsive drug delivery systems [95]. By incorporating stimuli-responsive polymers into the 4D-printed structures, drug release can be triggered by specific external stimuli, such as changes in temperature, pH, or enzyme activity. This enables controlled and targeted drug delivery, allowing for personalized treatment approaches and minimizing side effects. The dynamic nature of these materials ensures that the drug release adapts to the specific requirements of the patient, enhancing therapeutic efficacy and patient outcomes [96]. 

As mentioned before in this article, shape memory alloys (SMAs) are a unique class of metallic materials with intriguing non-linear properties, including pseudo-elasticity (PE), memory (MEM), and damping capacity, resulting from their high mechanical hysteresis and internal friction. Apicella et. al. adopted a bioinspired approach, drawing parallels between SMAs and intelligent materials that respond to thermal pulses with shrinkage, akin to muscle behavior [97]. SMAs underwent reversible deformations when subjected to external forces, exhibiting the potential for substantial shape changes in response to temperature fluctuations. The paper explored the concept of biomechanically inspired machines, inspired by pairs of antagonistic muscles found in skeletal systems. These machines leverage the SMA’s ability to generate reactive forces when recovering from shape changes, surpassing the initial force that caused the deformation [98]. This research not only provided a model for specific applications but also offers a versatile approach applicable to a wide range of adaptive uses, such as switching windows, intelligent shading systems, and more [99]. It marked a significant step in harnessing the potential of muscle-like acting Ni-Ti alloys, paving the way for innovative applications in adaptive systems and intelligent structures [100]. Table 4 summarizes the most crucial results in this field.

### 3.2. Robotics Field 

In robotics, biomimetic smart materials enable the development of self-transforming and adaptive robotic systems. By incorporating shape memory or stimuli-responsive be-haviors into 4D-printed robot components, these materials allow robots to change their shape, adjust their locomotion patterns, and respond to environmental conditions. Biomimetic smart materials offer advantages in terms of lightweight construction, energy efficiency, and the ability to perform complex tasks [101]. This opens up avenues for soft robotics, where robots can navigate unstructured environments, manipulate objects, and interact safely with humans [102]. 

One specific application of biomimetic smart materials in robotics is the develop-ment of soft robots with shape-changing capabilities. By utilizing 4D-printed materials that exhibit shape memory effects or stimuli-responsive behavior, robots can undergo controlled and reversible shape changes in response to external stimuli, such as tempera-ture, light, or humidity [103]. These materials allow for the creation of robots that can morph their shape, adjust their locomotion patterns, and adapt to the surrounding environment. Such robots have the potential to navigate complex and unstructured terrains, squeeze through tight spaces, and interact safely with humans [103]. 

Additionally, biomimetic smart materials enable the creation of robotic systems with artificial muscles that closely mimic the behavior and properties of natural muscles. These materials, often referred to as electroactive polymers (EAPs), can undergo mechanical actuation in response to electrical stimulation [104]. By integrating EAPs into 4D-printed structures, researchers can develop robots with soft and compliant actuators that enable natural-like movements and dexterity. This allows for safer human–robot interactions, precise manipulation of objects, and enhanced robot adaptability in dynamic environments [104]. 

Furthermore, biomimetic smart materials in 4D printing contribute to the develop-ment of robotic systems that exhibit self-healing and self-repairing capabilities. Taking inspiration from nature’s ability to heal and regenerate, researchers are exploring materi-als that can autonomously repair themselves when damaged [105]. By integrating self-healing mechanisms into the 4D-printed structures, robots can recover from damage, ensuring longer operational lifetimes and reducing maintenance requirements [105]. 

Also, according to relevant literature findings, Aversa et. al. investigated the intriguing material category of liquid crystals, substances that possess properties of both liquids and crystalline solids [106]. These phase-changing materials exhibited a remarkable ability to undergo different degrees of molecular alignment based on factors like temperature and electric potential. The study focused on the isothermal cycles of a Zr_44_-Ti_11_-Cu_10_-Ni_10_-Be_25_ metallic alloy in its metastable state, which was obtained by heating glassy samples. At elevated temperatures above the glass transition, the alloy displayed complex crystallization behaviors characterized by multiple and selective exothermic peaks, representing the crystallization of higher-mobility atoms within the alloy due to isothermal annealing. This experimental observation lead to an increase in the temperature required to induce glass metal relaxation, or vitreous transition [106]. Table 5 summarizes the most crucial results in this field.

### 3.3. Architectural Sector

Architectural applications of biomimetic smart materials in 4D printing offer the po-tential for adaptive and energy-efficient structures. Inspired by natural systems such as plant movements, biomimetic smart materials can be used to create building facades that respond to changes in temperature, light, or humidity, optimizing energy consumption and indoor comfort. These materials can also enable shape-changing architectural elements that adapt to environmental conditions, allowing for innovative and sustainable design approaches. 

One specific application lies in the development of adaptive building facades [107]. Biomimetic smart materials, such as those with shape memory effects or light-responsive behaviors, can be integrated into 4D-printed facades. These materials enable the creation of dynamic building envelopes that adapt their shape, permeability, or shading properties in response to changes in temperature, light intensity, or humidity [108]. By regulating the amount of solar radiation entering the building, these adaptive facades can optimize energy usage for heating, cooling, and lighting, thereby improving energy efficiency and reducing reliance on artificial systems [109]. 

Another application of biomimetic smart materials in architecture is the creation of self-assembling structures inspired by natural systems [110]. By incorporating materials that respond to specific triggers, such as temperature or moisture, 4D-printed components can autonomously assemble into complex structures. This approach mimics the growth and self-assembly mechanisms observed in natural organisms and offers opportunities for efficient and sustainable construction methods. Self-assembling structures can enable rapid deployment, reduce construction time and waste, and facilitate the creation of adaptable and reconfigurable spaces [111]. 

Biomimetic smart materials also contribute to the development of responsive archi-tectural elements, such as kinetic shading systems or ventilation systems [112]. By utilizing materials that change their shape or porosity in response to external stimuli, 4D-printed components can optimize airflow, daylighting, and thermal comfort within buildings [113]. These responsive elements can dynamically adapt to changing weather conditions, occupant preferences, or energy requirements, resulting in enhanced indoor environmental quality and reduced energy consumption [114]. 

Biomimetic smart materials can also be employed in the development of bioclimatic building envelopes that regulate internal temperature and humidity based on external climatic conditions [115]. These envelopes mimic the adaptive features found in natural systems, enabling buildings to self-regulate and maintain optimal indoor environmental conditions without excessive reliance on conventional heating, ventilation, and air conditioning systems. This promotes energy efficiency and occupant comfort while reducing the overall environmental footprint of the building. 

Leveraging biomimetic principles, 4D-printed architectural components can also be designed to manipulate natural light within a building, enhancing daylight penetration and distribution [116]. By incorporating light-responsive materials, such as those capable of controlling transparency or diffusing sunlight, architects can create spaces that offer a balance of natural illumination and privacy. This not only reduces the energy consumption associated with artificial lighting but also promotes a healthier and more productive indoor environment for building occupants. 

Additionally, biomimetic smart materials can be utilized to develop innovative architectural solutions that enhance acoustic performance within buildings [117]. By integrating materials inspired by natural sound-absorbing mechanisms, such as those found in certain plant structures or animal habitats, 4D-printed architectural elements can effectively mitigate noise pollution and reverberation. This contributes to the creation of quieter and more comfortable indoor spaces, fostering a conducive environment for work, relaxation, and social interaction. 

What is more, incorporating biomimetic smart materials in architectural designs enables the development of adaptive water management systems that mimic the self-regulating capabilities of natural water-retention mechanisms [118]. By integrating materials with responsive hydrophilic or hydrophobic properties, 4D-printed building components can efficiently manage water flow, reduce the risk of water-related damage, and promote sustainable water conservation practices. These adaptive systems contribute to the overall resilience and sustainability of the building infrastructure in the face of changing climate patterns and water scarcity challenges. Table 6 summarizes the most crucial results in this field. 

### 3.4. Aerospace Industry Sector

In the aerospace industry, biomimetic smart materials hold promise for the devel-opment of morphing structures and adaptive components [119]. Inspired by bird wings and plant leaves, 4D-printed biomimetic structures can change their shape during flight, op-timizing aerodynamic performance and reducing fuel consumption. The ability to create lightweight, flexible, and self-adaptive aerospace components has the potential to revolutionize aircraft design, leading to increased efficiency and improved maneuverability [120]. One specific application lies in the development of morphing wings and adaptive structures. By incorporating biomimetic smart materials into 4D-printed wing structures, aircraft can dynamically change their shape during flight to optimize aerodynamic performance [121]. These materials, inspired by bird wings and their ability to adjust their shape in response to varying flight conditions, allow for efficient control of lift, drag, and maneuverability. Morphing wings enable improved fuel efficiency, enhanced agility, and reduced noise emissions, contributing to more sustainable and advanced aircraft designs [122]. 

Biomimetic smart materials also find application in the creation of deployable structures and adaptive components. By utilizing 4D-printed materials that can undergo shape changes or actuation in response to external stimuli, such as temperature or electrical fields, aerospace engineers can design deployable systems that expand or fold during space missions or for compact storage during transport. These materials enable the development of lightweight and space-efficient structures that adapt to the operational requirements of spacecraft, satellites, or planetary rovers [123].

**Figure 7:**
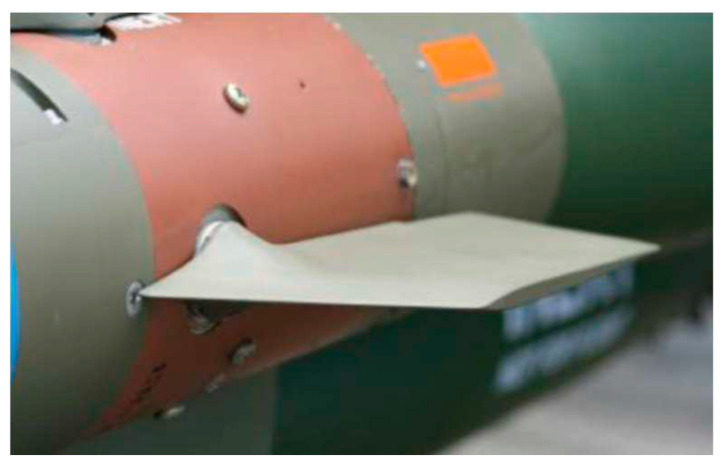
Utilization of SMA amorphous metals, where high precision parts/surfaces and volume production are requested in the military aerospace sector.

Figure 7, depicts a relevant application utilizing SMA amorphous metals, where high precision parts/surfaces and volume production are requested in the military aerospace sector [119].

Additionally, biomimetic smart materials in 4D printing contribute to the creation of self-healing and self-repairing aerospace components [124]. Inspired by the regenerative capabilities of natural organisms, researchers are exploring materials that can autonomously repair damages, such as microcracks or delamination. By incorporating self-healing mechanisms into 4D-printed structures, aircraft components can recover from structural damage, ensuring longer operational lifetimes, reducing maintenance costs, and enhancing safety in harsh environments [124].

Furthermore, the application of biomimetic smart materials extends to aerospace robotics and actuators. By integrating these materials into robotic systems and actuators, engineers can create lightweight, adaptable, and efficient mechanisms [125]. These biomimetic robotic systems can imitate the motions and behaviors of natural organisms, providing increased dexterity, flexibility, and efficiency in aerospace applications such as space exploration, satellite deployment, or maintenance operations [126]. Table 7 summarizes the most crucial results in this field.

Overall, the applications of biomimetic smart materials in 4D printing are vast and continually expanding. By harnessing nature’s design principles, these materials unlock new possibilities for tissue engineering, robotics, architecture, and aerospace applications. As research and development in this field progress, it is expected that we will see even more innovative applications emerge, pushing the boundaries of what is possible with additive manufacturing and biomimetic design.

## 4. Discussion

According to the aforementioned shape memory materials, including shape memory alloys (SMAs), shape memory polymers (SMPs), and electroactive polymers (EAPs), represent a diverse class of materials with unique properties that have garnered significant interest in the fields of engineering, materials science, and biomedical technology. Each of these materials demonstrates distinctive characteristics in terms of their responsiveness to external stimuli, actuation capabilities, and applications. In this context, a comprehensive comparison table, outlining the key features of these materials, their response mechanisms, actuation speeds, and varied applications, can be found in Table 8, highlighting their contributions to the advancement of modern technologies.

A complementary factor that might have notable impact in several aspects of shape memory biomimetic materials is texture. Texture, in the context of biomimetic shape memory materials, refers to the surface characteristics and structural features of the mate-rial. The effect of texture on the shape memory of biomimetic shape memory materials can be significant and is often a subject of research and development in materials science [126]. Firstly, the texture of biomimetic shape memory materials can affect their mechanical properties [127]. For example, surface roughness or patterns inspired by natural structures can influence the material’s stiffness, flexibility, and overall mechanical performance. These properties, in turn, can impact the material’s ability to undergo shape memory transformations. Secondly, the texture of biomimetic materials can be designed to enhance shape fixity and recovery [128]. Mimicking the microstructures found in certain natural materials, such as muscle fibers or collagen, can contribute to improved shape memory performance. These textures may guide and control the deformation and recovery processes in the material. Also, texture can affect surface interactions, such as adhesion and friction, which play a role in the shape memory behavior of biomimetic materials [129]. Optimizing the surface texture can reduce friction during shape recovery, allowing for smoother and more efficient transitions between different shapes. Some biomimetic shape memory materials are responsive to temperature changes. Texture can influence how these materials respond to temperature variations [130]. For instance, certain surface textures may enhance heat transfer, affecting the speed and efficiency of the shape memory transition at different temperatures. In addition, the texture of biomimetic materials is crucial in applications where biocompatibility is essential, such as in medical implants [131]. Mimicking the texture of natural tissues can improve integration with biological systems, and this, in turn, can impact the overall performance and stability of shape memory materials in biological environments. Lastly, texture can be strategically engineered on the surface of biomimetic shape memory materials to create “smart surfaces” that respond to external stimuli [132]. This can include designing textures that respond to specific biochemical signals or environmental conditions, expanding the range of applications for these materials. 

While biomimetic smart materials offer exciting opportunities for 4D printing and the creation of self-transforming objects, several challenges need to be addressed to fully harness their potential. One significant challenge lies in the precise control of material properties and responses. Biomimetic smart materials often exhibit complex behaviors that need to be accurately replicated and controlled in the manufacturing process. Achieving consistent and predictable shape changes, as well as appropriate responses to stimuli, requires a deep understanding of material science, biology, and engineering. Researchers ensure trigger reliability and specificity for external stimuli-induced changes in biomimetic smart materials through meticulous material design and testing methodologies. They focus on engineering materials with precise molecular or structural responsiveness to intended stimuli, conducting rigorous testing to validate trigger reliability and specificity. This process involves thorough experimentation to verify that the material responds accurately and consistently to the designated stimuli while remaining inert to other environmental factors. By fine-tuning material composition, molecular structures, and fabrication techniques, researchers strive to achieve a high degree of reliability and specificity in triggering desired changes within biomimetic smart materials, ensuring their efficacy in real-world applications. 

The integration of biomimetic smart materials into widespread industrial use faces challenges concerning scalability and compatibility with existing 4D printing techniques. While initial successes in laboratory settings showcase the potential, transitioning these materials from small-scale prototypes to large-scale production presents significant hurdles. To accommodate biomimetic smart materials effectively, adaptations in manufacturing processes are crucial. This involves optimizing existing techniques or developing new methodologies tailored for industrial-scale applications while preserving the material’s functionality and performance. This necessitates a delicate balance between scalability, cost-effectiveness, and maintaining high-quality production standards, posing an ongoing area of research and innovation within the field. Changes needed to accept biomimetic smart materials on a larger scale encompass advancements in printing technologies, such as improved precision and speed, while ensuring materials’ compatibility with mass production methods. Additionally, standardization of material properties, better characterization methods, and advancements in post-processing techniques are imperative to streamline integration into industrial workflows. Moreover, research efforts focus on developing novel material formulations that offer enhanced manufacturability, durability, and responsiveness to stimuli, facilitating their seamless adoption within existing 4D printing frameworks. 

Another challenge lies in the long-term durability and stability of biomimetic smart materials. Ensuring that these materials retain their functionality over extended periods is crucial for their practical viability in real-world applications. Factors such as degradation, fatigue, and aging can impact their performance and longevity. Exploring ways to enhance the durability and stability of these materials, as well as understanding their long-term behavior under different environmental conditions, is essential for their successful implementation. 

Additionally, the development of biomimetic smart materials for 4D printing requires multidisciplinary collaboration among experts from various fields, including materials science, biology, engineering, and design. Bridging the knowledge gap and fostering effective communication among these disciplines is vital to fully capitalize on the potential of biomimetic smart materials. Collaborative efforts can accelerate advancements and address the challenges associated with these materials, leading to transformative applications in various industries. 

The incorporation of biomimetic smart materials as raw materials in 4D printing is poised to play a pivotal and transformative role in the pursuit of environmental sustaina-bility and the mitigation of our ecological footprint. This innovative fusion of biomimic-ry-inspired materials and cutting-edge 4D printing technology presents an unprecedented opportunity to address pressing environmental challenges and usher in a sustainable and adaptive future. 

Biomimicry, as a well-established discipline grounded in emulating nature’s intri-cate solutions, serves as the foundational framework for the development of materials imbued with remarkable attributes, including adaptability, responsiveness, and self-transformation. The resulting symbiosis between advanced manufacturing techniques and nature-inspired materials holds immense potential to yield objects capable of dynamic responses to environmental stimuli. 

By harnessing the power of biomimetic smart materials in 4D printing, we are pre-sented with a unique opportunity to address several critical environmental goals. For example, regarding the mitigation of waste generation, the utilization of biomimetic mate-rials allows for the creation of objects that can adapt and evolve, reducing the need for disposable products. This, in turn, minimizes waste generation and contributes to a circular economy. Thus, energy consumption can be minimized while the self-transformative and adaptive capabilities of these materials can lead to the development of energy-efficient products. This translates to reduced energy consumption throughout the lifecycle of these objects. 

Also, the environmental footprint can be reduced due to the biomimetic materials’ innate ability to self-repair and adapt promotes the longevity of products, reducing the need for constant replacements. This leads to a decreased environmental footprint associ-ated with manufacturing, transportation, and disposal. 

In this context, a sustainable future emerges wherein inanimate objects transcend their static nature and evolve into dynamic entities capable of self-repair, adaptation, and responsive behaviors in harmony with their surroundings. The consolidation of biomim-icry and 4D printing techniques establishes a seminal precedent for a profound reconfig-uration of contemporary approaches to design, manufacturing, and ecological steward-ship. 

Addressing these challenges will require continued research, innovation, and technological advancements. By overcoming these hurdles, biomimetic smart materials can truly shape the future of self-transforming objects and unlock new possibilities in fields such as healthcare, robotics, architecture, and aerospace, transforming the way we design, manufacture, and interact with adaptive materials and structures. 

## 5. Conclusions

In conclusion, the incorporation of biomimetic smart materials in the greater technological field of 4D printing holds significant promise in influencing the trajectory of self-transforming entities. The capacity of these materials to demonstrate dynamic responses, adaptability, and self-transformation presents novel opportunities across diverse industries. The incorporation of biomimetic smart materials exhibits significant potential for pioneering applications across a range of fields, including healthcare, robotics, architecture, and aerospace applications. The fusion of biomimetic smart materials with 4D printing technology offers a transformative path towards environmental sustainability. Drawing inspiration from nature’s intricate designs, this integration empowers materials with adaptability and self-transformation capabilities, revolutionizing manufacturing paradigms. These biomimetic materials not only reduce waste generation but also minimize energy consumption and the overall environmental footprint. By enabling inanimate objects to evolve, self-repair, and adapt to their surroundings, this synergy pioneers a sustainable future. Despite the presence of challenges such as material control, scalability, and durability, ongoing research and technological advancements are being undertaken to tackle these concerns. Researchers are actively focusing on enhancing material properties, improving scalability, and ensuring long-term durability to facilitate broader industrial applications. As the investigation and enhancement of biomimetic smart materials in 4D printing continue, we are facilitating the progression of groundbreaking developments in personalized medicine, adaptable robotics, sustainable architecture, and other related fields. These materials possess promising potential to significantly transform industries and influence the advancement of self-transforming objects, thereby offering various enhancements in a number of fields, ultimately contributing to a more sustainable and technologically advanced future. 

## Figures and Tables

**Table 1:** Most common SMAs, their applications and their most prominent features.

SMA Type	Applications	Strength	Cost-Effectiveness	Biocompatibility
Nitinol (NiTi) [66]	Medical devices, eyeglass frames, robotics	High	Moderate	Good
Cu-Zn-Al [59]	Actuators, robotics, aerospace	Moderate	Moderate	Poor
Ni-Ti-Pd [60]	Aerospace, medical implants	High	Moderate	Good
Fe-Pt [68,69]	Actuators, sensors, robotics	High	High	Poor
Cu-Al-Ni [67]	Robotics, automotive	Moderate	Moderate	Poor
Ni-Al-Mn [65]	Actuators, medical devices	High	Moderate	Good

**Table 4:** Key findings in the use of biomimetic materials in the regenerative medicine field.

Key Findings and Applications	Description
Biomimetic scaffolds [89,90]	Introduction of biomechanical scaffolds based on nanodiamond-filled hydrophilic polymer matrix
Unique material properties [88]	Novel biomaterials with special mechanical and biological properties for advanced biomedical applications
Enhanced mechanical strength [91]	Hybrid nanocomposites with significantly improved mechanical strength compared to traditional hydrogels
Orthopedic applications [92]	Biomimetic implants find potential applications in orthopedic areas like the knee, ankle, hip, shoulder, and orthopedic column
Surgical oncology [93,94]	Supports bone regeneration following tumor elimination interventions in surgical oncology
Odonto-stomatological implants [98]	Development of bioactive odonto-stomatological implants using hybrid ceramic–polymeric biomimetic material
Bone remodeling stimulation [94]	Emphasis on creating bioactive scaffolding interfaces that stimulate healthy bone remodeling and growth
Biomechanical compatibility [89,90]	Use of mechanically compatible hybrid hydrogels to enhance prosthesis adaptation and replicate biomechanical functions of cartilage and ligaments

**Table 5:** Key findings in the use of biomimetic materials in the robotics field.

Key Findings and Applications	Description
Soft robots with shape-changing abilities [103]	Utilization of 4D-printed materials exhibiting shape memory effects or stimuli-responsive behavior, enabling robots to undergo controlled and reversible shape changes in response to external stimuli like temperature, light, or humidity. This allows for navigation of complex terrains, squeezing through tight spaces, and safe human interaction.
Robotic systems with artificial muscles [104]	Integration of electroactive polymers (EAPs) into 4D-printed structures, enabling the development of robots with soft and compliant actuators that mimic the behavior and properties of natural muscles. This facilitates safer human–robot interactions, precise manipulation of objects, and enhanced robot adaptability in dynamic environments.
Robotic systems with self-healing capabilities [105]	Exploration of materials that can autonomously repair themselves when damaged, ensuring longer operational lifetimes and reducing maintenance requirements. Integration of self-healing mechanisms into 4D-printed structures allows robots to recover from damages and enhance durability.
Application of liquid crystals in robotics [106]	Investigation of liquid crystals, substances possessing properties of both liquids and crystalline solids. Research focuses on the isothermal cycles of specific metallic alloys, studying their behavior under different temperature and electric potential conditions, leading to improved understanding of their properties and applications in robotics.
Integration of biomimetic smart materials for sensing and feedback [106]	Incorporation of biomimetic materials for sensing and feedback mechanisms in robots, allowing them to interact with their environment more effectively. These materials enable the development of robots with enhanced perception capabilities, facilitating tasks such as object manipulation, navigation, and adaptive responses to external stimuli.

**Table 6:** Key findings in the use of biomimetic materials in the architectural field.

Key Findings and Applications	Description
Adaptive Building Facades [107,108,109]	Integration of biomimetic smart materials, such as those with shape memory effects or light-responsive behaviors, into 4D-printed facades, enabling the creation of dynamic building envelopes that adapt their shape, permeability, or shading properties in response to changes in temperature, light intensity, or humidity.
Self-Assembling Structures [110,111]	Incorporation of materials that respond to specific triggers, such as temperature or moisture, allowing 4D-printed components to autonomously assemble into complex structures. This mimics the growth and self-assembly mechanisms observed in natural organisms, promoting efficient and sustainable construction methods and facilitating the creation of adaptable and reconfigurable spaces.
Responsive Architectural Elements [112,113,114]	Utilization of materials that change their shape or porosity in response to external stimuli in 4D-printed components, optimizing airflow, daylighting, and thermal comfort within buildings. These responsive elements dynamically adapt to changing weather conditions, occupant preferences, or energy requirements, resulting in enhanced indoor environmental quality and reduced energy consumption.
Bioclimatic Building Envelopes [115]	Development of building envelopes that regulate internal temperature and humidity based on external climatic conditions, mimicking adaptive features found in natural systems. These envelopes enable buildings to self-regulate and maintain optimal indoor environmental conditions, promoting energy efficiency and occupant comfort while reducing the overall environmental footprint.
Natural Light Optimization [116]	Design of 4D-printed architectural components that manipulate natural light within a building, enhancing daylight penetration and distribution. Incorporation of light-responsive materials enables the creation of spaces that balance natural illumination and privacy, reducing energy consumption associated with artificial lighting and promoting a healthier and more productive indoor environment for occupants.
Enhanced Acoustic Performance [117]	Implementation of innovative architectural solutions that enhance acoustic performance within buildings. Integration of materials inspired by natural sound-absorbing mechanisms effectively mitigates noise polution and reverberation, creating quieter and more comfortable indoo spaces conducive to work, relaxation, and social interaction.
Adaptive Water Management Systems [118]	Utilization of biomimetic smart materials to develop adaptive water management systems that mimic the self-regulating capabilities of natural water-retention mechanisms. Integration of materials with responsive hydrophilic or hydrophobic properties enables efficient water flow management, reduces the risk of water-related damage, and promotes sustainable water conservation practices, enhancing the resilience and sustainability of building infrastructure.

**Table 7:** Key findings in the use of biomimetic materials in the aerospace industry field.

Key Findings and Applications	Description
Morphing wings and adaptive structures [120,121,122]	Integration of biomimetic smart materials into 4D-printed wing structures, enabling aircraft to dynamically change their shape during flight to optimize aerodynamic performance. Inspired by bird wings, these materials allow for efficient control of lift, drag, and maneuverability, leading to improved fuel efficiency, enhanced agility, and reduced noise emissions, contributing to more sustainable and advanced aircraft designs.
Deployable structures and adaptive components [123]	Utilization of 4D-printed materials capable of shape changes or actuation in response to external stimuli, such as temperature or electrical fields, to design deployable systems that expand or fold during space missions or for compact storage during transport. These materials enable the development of lightweight and space-efficient structures that adapt to the operational requirements of spacecraft, satellites, or planetary rovers.
Self-healing and self-repairing aerospace components [124]	Exploration of materials capable of autonomously repairing damages, such as microcracks or delamination, inspired by the regenerative capabilities of natural organisms. Incorporation of self-healing mechanisms into 4D-printed structures enables aircraft components to recover from structural damage, ensuring longer operational lifetimes, reducing maintenance costs, and enhancing safety in harsh environments.
Aerospace robotics and actuators [125,126]	Integration of biomimetic smart materials into robotic systems and actuators, allowing engineers to create lightweight, adaptable, and efficient mechanisms. These biomimetic robotic systems can imitate the motions and behaviors of natural organisms, providing increased dexterity, flexibility, and efficiency in aerospace applications such as space exploration, satellite deployment, or maintenance operations.

**Table 8:** Comparative analysis of shape memory materials by exploring the properties and applications of SMAs, SMPs, and EAPs.

Property	SMAs	SMPs	EAPs
Material type	Metal alloys	Polymers	Polymers
Response mechanism	Temperature-driven	Temperature-driven	Electrically-driven
Actuation speed	Fast	Moderate to fast	Fast
Reversibility	Fully reversible	Fully or partially reversible	Fully reversible
Applications	Biomedical devices, aerospace components	Biomedical applications, soft robotics	Robotics, artificial muscles, haptic devices
Cost	Relatively high	Moderate	Moderate to high

## Data Availability

No new data were created or analyzed in this study. Data sharing is not applicable to this article.
